# Comparative pharmacokinetics and urinary excretion of arsenic and mercury after oral administration of realgar, cinnabar and AnGongNiuHuang Pill to rats

**DOI:** 10.3389/fphar.2022.967608

**Published:** 2022-08-30

**Authors:** Xiao Wu, Zeling Zhong, Kuangmin Lin, Xinhe Liu, Zhichao Wu, Zitian Liu, Yongming Li

**Affiliations:** School of Medicine and Holistic Integrative Medicine, Nanjing University of Chinese Medicine, Nanjing, China

**Keywords:** realgar, cinnabar, AnGongNiuHuang pill, arsenic, mercury, pharmacokinetics, urinary excretion, hydride-generation atomic fluorescence spectrometry

## Abstract

Realgar- and cinnabar-containing AnGongNiuHuang Pill (AGNHP) is widely used for treating encephalopathy syndrome. However, it raises great safety concerns due to the adverse effects reported by arsenic or mercury poisoning. Although AGNHP has been generally recognized, little is known about the metabolism of arsenic and mercury and their resulting potential health risk *in vivo*. Thus, comparative pharmacokinetics and urinary excretion of arsenic and mercury were conducted in rats after oral administration of realgar, cinnabar and AGNHP, respectively. The contents of arsenic and mercury in rat blood and urine were determined by hydride-generation atomic fluorescence spectrometry (HG-AFS) after wet digestion. AGNHP significantly reduced the absorption of arsenic in blood and promoted urinary arsenic excretion. Whereas, it increased the blood mercury absorption and reduced urinary mercury excretion. No significant toxicity was observed in the clinical dose range of AGNHP. However, excessive exposure to arsenic and mercury may still pose risks especially by long-term or excessive medication. The results are helpful for the rational clinical applications of realgar- and cinnabar-containing TCMs.

## Graphical Abstract

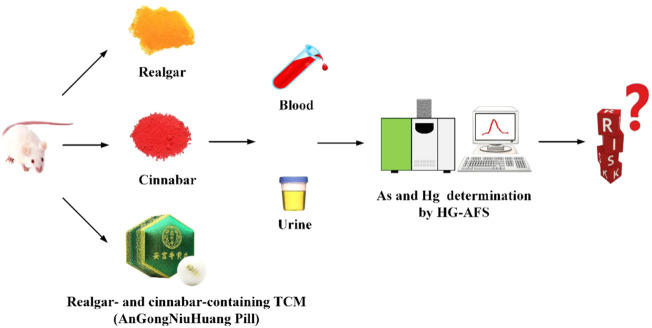

## Introduction

Heavy metals are dense chemicals found naturally on a daily life basis. Heavy metals are classified as micronutrients or toxicants according to their toxicity (Kim et al., 2019). Arsenic and mercury are the most common toxic heavy metals in the environment, which exert toxic effects even at low concentrations. Ingestion, inhalation or dermal absorption of arsenic may result in gastrointestinal syndrome, neurodegenerative disorders, cardiovascular disease, diabetes, skin lesions, and cancers of the lung, kidney and bladder (Rehman et al., 2018). Mercury is a deadly neurotoxin substance, and its exposure is mainly through inhalation of mercury vapor and ingestion of organic mercury. Mercury toxicity is associated with neurological and immune dysfunction, pneumonitis, acute necrotizing bronchitis, tremor, gastrointestinal disturbance and brain damage (Rahman and Singh 2019; Tsoi et al., 2019). Since heavy metals can disturb the body’s metabolic functions in various ways, the WHO and the Food and Agriculture Organization (FAO) regulated the maximum weekly intake levels for arsenic and mercury (Sarma et al., 2011).

Interestingly, minerals containing large amounts of arsenic and mercury are intendedly added in numerous Traditional Chinese Medicine (TCM) formulas since ancient China, which has proved to have remarkable effects on various diseases. Realgar and cinnabar are the most extensively used arsenic- and mercury-containing mineral medical materials, respectively. Realgar (As_2_S_2_) has been widely used to treat carbuncles, malaria, psoriasis, convulsive epilepsy and parasitic infections (Wu et al., 2020). In recent years, realgar is used as an alternative to arsenic trioxide in treating acute promyelocytic leukemia (Lou et al., 2021). Cinnabar (HgS) has sedative and hypnotic effects, which is used to treat insomnia, dreaminess, epilepsy and infantile convulsions (Yang et al., 2020). In the 2020 edition of Chinese pharmacopeia, there are 38 (38/1607, 2.4%) and 74 (74/1607, 4.6%) types of TCMs containing realgar and cinnabar, respectively, among which 26 (26/1607, 1.6%) types contain both realgar and cinnabar. AnGongNiuHuang Pill (AGNHP) is the best-known realgar- and cinnabar-containing TCM preparation for treating stroke, encephalitis, meningitis, hematencephalon, convulsion, hyperpyrexia and coma (Zhang et al., 2021). It consists of realgar (10%), cinnabar (10%), Hyriopsis cumingii (Lea), Bovis Calculus, Powered Buffalo Horn Extract, natural or artificial Moschus, Coptis chinensis Franch., Dryobalanops aromatica C. F. Gaertn., Gardenia jasminoides J. Ellis, Curcuma aromatica Salisb. and Scutellaria baicalensis Georgi.

Because realgar and cinnabar are water-insoluble, their toxicities should not be deemed as toxic as the equivalent inorganic arsenic or mercury. Our previous studies had confirmed that realgar- or cinnabar-containing TCMs were relatively safe in the therapeutic dose range (Wu et al., 2018; Lu et al., 2020; Wu et al., 2020; Wu et al., 2022). However, poisoning cases caused by overdose or long-term use of realgar- or cinnabar-containing TCMs have been reported occasionally (Wu and Deng 2013; Wu et al., 2013; Chang et al., 2018). AGNHP is forbidden in foreign countries because it contains excessive levels of arsenic and mercury (Xia et al., 2018). Since realgar and cinnabar are the essentially active components of AGNHP (Xia et al., 2018; Tsoi et al., 2019), the safety of AGNHP has aroused great concerns among the public. Previous studies reported that AGNHP was protective against cinnabar- and realgar-induced hepatic and renal damage (Li et al., 2018; Wang et al., 2021). Whereas, the interaction between arsenic and mercury in AGNHP and their resulting potential health risk have not been well studied *in vivo*. Therefore, the metabolisms of arsenic and mercury after oral administration of AGNHP need to be urgently addressed in a biological system.

In this study, the safety of AGNHP was evaluated through the interaction of arsenic and mercury based on pharmacokinetics and urinary excretion. The metabolic differences of arsenic and mercury were compared in rats after single oral gavage of realgar, cinnabar and AGNHP, respectively. Arsenic and mercury in rat blood and urine were determined by hydride-generation atomic fluorescence spectrometry (HG-AFS) after wet digestion. The study aims to provide guidance for the safety and clinical usage of realgar- and cinnabar-containing TCMs.

## Materials and methods

### Chemicals and reagents

AGNHP (Batch No.19011426, 3 g/pill) was purchased from Beijing Tongrentang Technologies Co., Ltd. (Beijing, China). Water lapped realgar (Batch No.160319, purity of 92.86%) was obtained from Sanmenxia Yuhuangshan Pharmaceutical Co., Ltd. (Henan, China). Water lapped cinnabar (Batch No. 20181201, purity of 96.23%) was bought from Fenghuang Hongfei cinnabar Pharmaceutical Co., Ltd. (Hunan, China). Nitric acid, sulphuric acid, hydrochloric acid, perchloric acid, potassium borohydride, potassium dichromate, potassium hydroxide, potassium hydroxide, thiourea, ascorbic acid and sodium carboxymethyl cellulose (CMC-Na) were obtained from Sinopharm Chemical Reagent Co., Ltd. (Shanghai, China). Heparin sodium was supplied by Beijing Solarbio Science and Technology Co., Ltd. (Beijing, China). Arsenic and mercury standard solutions (1000 mg/L) were obtained from o2si smart solutions Co., Ltd. (Charleston, United States) and Guobiao Testing and Certification Co., Ltd. (Beijing, China), respectively. Deionized water was produced by a PCDX-F10 water purification system purchased from Pincheng Science and Technology Co., Ltd. (Chengdu, China).

### Animals

Adult male Sprague-Dawley rats (180–200 g) were obtained from Xipuer-Bikai Laboratory Animal Co., Ltd (Shanghai, China). Rats were raised in the animal laboratory of Nanjing University of Chinese Medicine (Nanjing, China) at a temperature of (22 ± 3)°C and humidity of 40–60%. Rats were acclimatized for 1 week and fed sterile feed and water *ad libitum*. All animals were fasted overnight before the study.

The animal experiments were performed under animal use guidelines and approved by the Committee on Animal Research and Ethics of the Nanjing University of Chinese Medicine.

### Quality control of TCMs

The quality of realgar, cinnabar and AGNHP was controlled following the 2020 edition of Chinese Pharmacopeia. The arsenic and mercury contents of TCMs were determined by the same procedure as previously reported (Lu Y. T. et al., 2017; Wu et al., 2022). Each AGNHP contained an average of 84 mg of mercury and 122 mg of arsenic. The average contents of arsenic and mercury were 677 mg/g and 860 mg/g in realgar and cinnabar, respectively.

### Drug administration and sample collection

AGNHP was cut into pieces, and realgar and cinnabar were grounded into fine powders and suspended in 0.5% CMC-Na solution for administration. A total of 72 rats were randomly divided into 6 groups with 12 rats in each group. For each group, 7 rats were used for pharmacokinetic study, and the other 5 were placed individually in metabolic cages for urine excretion study. Rats were orally administrated with low and high doses of realgar, cinnabar and AGNHP at a single dose, respectively. The low and high doses of AGNHP were 308.5 and 1542.5 mg/kg for rats, equivalent to 1 and 5 times the human therapeutic dose, respectively. The corresponding low and high doses of realgar (18.0 and 90.0 mg/kg) and cinnabar (10.4 and 52.1 mg/kg) were calculated by equal amounts of realgar and cinnabar in AGNHP, respectively.

Blood samples of approximately 0.15 ml were collected from the rat angular vein into heparinized polythene tubes at 0, 0.25, 0.5, 0.75, 1, 2, 4, 8, 12, 24 and 48 h after dosing, respectively. Urine samples were collected at 0–4, 4–8, 8–12 and 12–24 h after dosing. The volumes of urine samples were measured and recorded. All the biological samples were stored at −80°C for further analysis.

### Sample preparation and analytical methods

An aliquot of 0.1 ml of blood or urine samples were pretreated with a modified fast Kjeldahl digestion for arsenic determination as our previously reported (Wu et al., 2018; Wu et al., 2022). After digestion, thiourea and ascorbic acid were added to reduce As^5+^ to As^3+^ before HG-AFS analysis (Wu et al., 2018; Wu et al., 2022). For mercury determination in biological samples, 0.1 ml of blood or urine samples were placed in a Kjeldahl flask. After adding 4 ml of nitric acid and 1 ml of perchloric acid, the Kjeldahl flask was capped with a funnel and left overnight at room temperature. Then the mixture was digested on a hotplate until a clear and transparent solution was produced. The digested solution was cooled and transferred to a 10 ml volumetric flask with 5% HNO_3_ (v/v)-0.05% K_2_Cr_2_O_7_ (w/v) solution and diluted to volume. Samples beyond the linear ranges of arsenic and mercury were diluted to appropriate concentrations for analysis.

The calibration standards were prepared with an arsenic standard solution in the concentration range from 5 to 100 ng/ml in the same way as previously reported (Wu et al., 2018; Wu et al., 2022). Series of mercury standards were prepared by appropriate dilution of mercury stock solution with 5% HNO_3_ (v/v)-0.05% K_2_Cr_2_O_7_ (w/v) solution in the linear range from 0.2 to 1.0 ng/ml. Sensitive and simple methods were developed and validated for arsenic and mercury determination in biological samples. The concentrations of arsenic and mercury in rat blood and urine were determined by a 9750 HG-AFS system (Haiguang Instrument Co., Ltd., Beijing, China). The analytical parameters are displayed in Table 1.

**Table 1:** HG-AFS parameters for arsenic and mercury determination.

**HG-AFS Parameters**	**Arsenic**	**Mercury**
Detection wavelength (nm)	193.7	253.7
Lamp current (mA)	60	30
Negative high-voltage (V)	250	280
Atomizer height (mm)	8	10
Carrier gas (mL/min)	Argon, 400	
Auxiliary gas (mL/min)	Argon, 1000	
Analysis period (s)	16	25
Delay period (s)	4	6
Measurement mode	Peak area	
Carrier solution	5% HCl (v/v)	5% HNO_3_ (v/v)
Reducing agent	2% KBH_4_ in 0.5% KOH (w/v)	0.1% KBH_4_ in 0.2% KOH(w/v)

### Statistical analysis

The pharmacokinetics and urinary excretion data are presented as mean ± standard deviation (SD) and mean ± standard error of the mean (SEM), respectively. The pharmacokinetic parameters were calculated by non-compartmental analysis (NCA) using Phoenix WinNonLin 7.0 (Pharsight Corporation, California, United States). The independent *t*-test was used to compare the differences between the two groups. The statistical analysis was performed using GraphPad Prism 8.0 (GraphPad Software, Inc., San Diego, CA). Differences were considered statistically significant at *p* < 0.05.

## Results and discussion

### Method validation

The optimized methods were validated for the determination of arsenic and mercury in biological samples in terms of linearity, limit of detection (LOD), precision, accuracy and stability. Arsenic and mercury showed good linearity in the range of 5–100 ng/ml and 0.2–1.0 ng/ml, respectively, with the correlation coefficients greater than 0.999. LOD was calculated by the ratio of 3SD and the slope of the calibration curve after 11 blank injections. LODs of arsenic and mercury were 0.0019 and 0.017 ng/ml, respectively. The precision and accuracy were determined from five replicate determinations. The precisions were all within 10%, and the accuracies were in the range of 95–111%. Arsenic and mercury in biological samples were transformed into the oxidation forms of As^5+^ and Hg^2+^ after digestion, respectively, which was independent of the freeze–thaw cycles, storage temperature and time. Therefore, the stability of arsenic and mercury were evaluated by the digestion solutions at room temperature for 24 and 4 h, respectively. Arsenic and mercury were stable when stored at room temperature with RSDs less than 10%.

### Pharmacokinetic study

**FIGURE 1:**
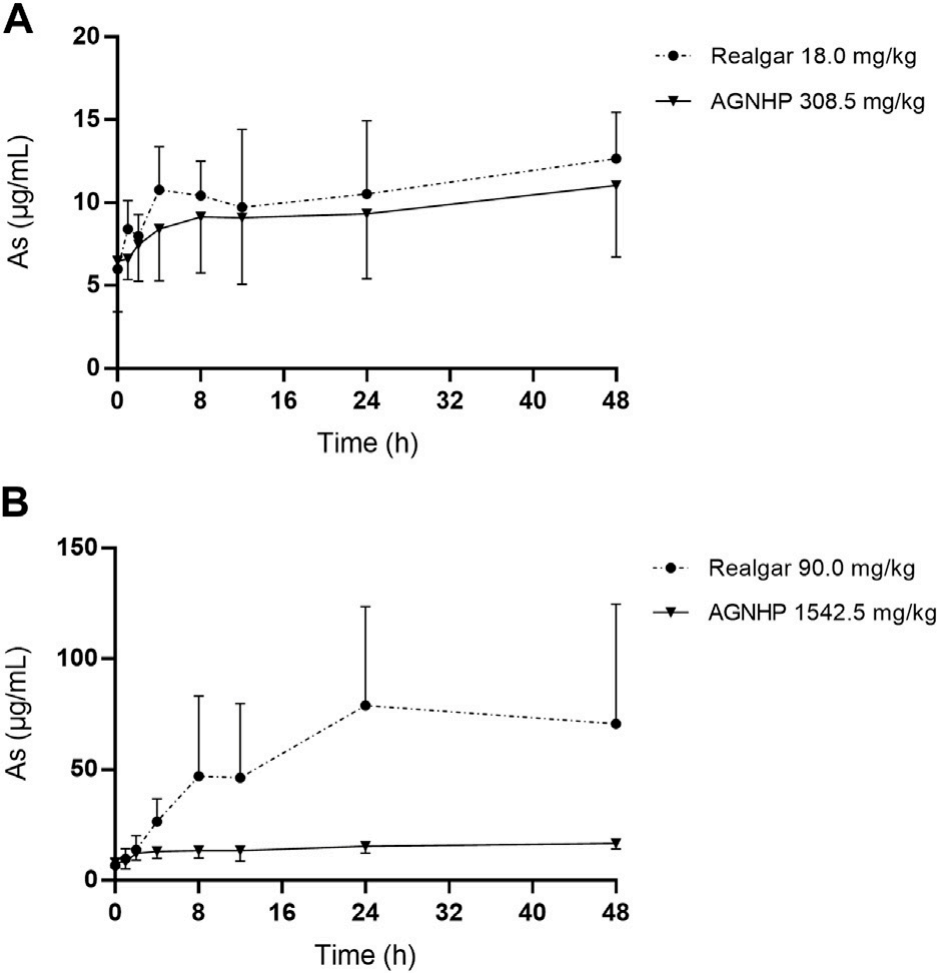
Blood concentration-time profiles of arsenic in rats after oral administration of realgar and AGNHP.

The mean blood concentration-time curves of arsenic in rat blood after oral administration of realgar and AGNHP are shown in Figure 1. The pharmacokinetic parameters of C_max_ (peak concentration), T_max_ (peak time), AUC (area under the curve) and MRT (mean residence time) are displayed in Table 2. It was observed that blood arsenic showed a dose-dependent increase in realgar and AGNHP groups, respectively. As the dose increases, the increase of blood arsenic in AGNHP groups was not as obvious as that of realgar. The blood arsenic concentration profiles of rats after oral administration of AGNHP were decreased compared to the corresponding realgar groups, which displayed a significant difference at high doses. The C_max_ of realgar and AGNHP was not significantly different at low doses due to the limited arsenic absorption in blood. Since the arsenic concentration did not decrease 48 h after dosing, T_1/2_ could not be calculated accurately. The prolonged T_max_ and MRT showed a slow absorption of arsenic in blood after oral administration of realgar and AGNHP to rats. Slow absorption and elimination of arsenic were observed in rat blood after dosing. The AUC of arsenic in AGNHP group showed a decrease compared to the corresponding realgar groups, which was consistent with the trend of C_max_. Overall, AGNHP significantly influenced the pharmacokinetic behaviors of arsenic in realgar.

**Table 2:** Pharmacokinetic parameters of arsenic in rats after oral administration of realgar and AGNHP.

	**Realgar (18.0 mg/kg)**	**AGNHP (308.5 mg/kg)**	**Realgar (90.0 mg/kg)**	**AGNHP (1542.5 mg/kg)**
C_max_ (μg/mL)	14.3 ± 3.6	11.9 ± 4.4	85.8 ± 50.6	17.9 ± 1.8^**^
T_max_ (h)	22.9 ± 18.0	27.4 ± 20.2	32.6 ± 15.0	29.4 ± 18.9
AUC_0-48h_ (h·μg/mL)	516 ± 128	456 ± 165	2942 ± 1718	709 ± 107^**^
MRT (h)	25.7 ± 1.5	25.6 ± 1.4	27.3 ± 2.7	25.7 ± 1.4

Significant difference was found between AGNHP and the corresponding realgar dose groups (^**^ *p*<0.01).

Previous studies had confirmed that realgar-containing TCMs significantly reduced the total blood arsenic exposure present in realgar, which might be attributed to the co-existing ingredients in realgar-containing TCMs (Wu et al., 2018; Wu et al., 2020). The reduced toxicity of realgar-containing TCMs might be related to the reduction of arsenic absorption by compatibility (Wang et al., 2021). Blood arsenic concentration reflected the absorption and was an important indicator of arsenic poisoning. As reported previously, a patient died of arsenic poisoning with a blood arsenic concentration of 21.1 μg/ml, and the fatal blood arsenic concentration was 0.16–41 μg/ml (Lech and Trela 2005; Lu et al., 2019). The blood arsenic concentration in poisoned rats was 144.00–166.11 μg/ml (Sayed et al., 2015). No significant toxicity occurred in rats after 30 days treatment of realgar, and the blood arsenic concentration was 68.62 μg/ml (Yi et al., 2019; Yi et al., 2020). Consequently, there is no risk of arsenic poisoning when taken AGNHP at clinical doses.

**FIGURE 2:**
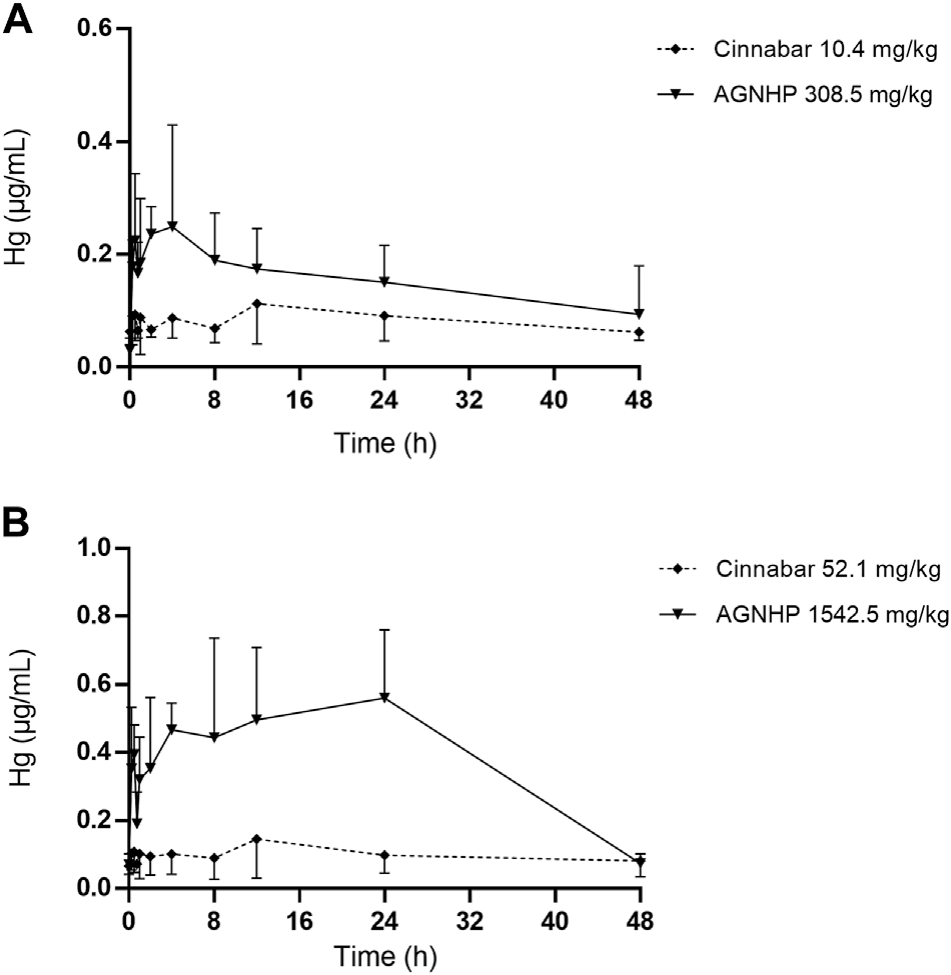
Blood concentration-time profiles of mercury in rats after oral administration of cinnabar and AGNHP.

The blood concentration–time profiles of mercury in rats after oral administration of cinnabar and AGNHP are shown in Figure 2. The blood mercury absorption was much lower than arsenic after oral administration of cinnabar and AGNHP to rats. Blood mercury presented a dose-dependent increase in cinnabar and AGNHP groups, respectively. Interestingly, AGNHP significantly increased the blood mercury absorption compared to the corresponding cinnabar groups, which was in contrast to the results of arsenic aforementioned. The C_max_ and AUC of mercury in AGNHP group showed a significant increase compared to the corresponding cinnabar groups (Table 3). The T_max_ of mercury revealed that mercury was slowly absorbed in rat blood, and it was slightly shorter than arsenic. Compared with the AGNHP groups, the MRT of mercury was extended after cinnabar treatment, which presented a significant difference in high doses.

**Table 3:** Pharmacokinetic parameters of mercury in rats after oral administration of cinnabar and AGNHP.

	**Cinnabar (10.4 mg/kg)**	**AGNHP (308.5 mg/kg)**	**Cinnabar (52.1 mg/kg)**	**AGNHP (1542.5 mg/kg)**
C_max_ (μg/mL)	0.15 ± 0.053	0.36 ± 0.13^**^	0.21 ± 0.088	0.70 ± 0.19^****^
T_max_ (h)	13.0 ± 8.2	2.0 ± 1.5^**^	12.7 ± 11.0	11.5 ± 9.3
AUC_0-48h_ (h·μg/mL)	4.0 ± 1.4	7.6 ± 3.0^*^	4.8 ± 2.0	19.1 ± 5.5^****^
MRT (h)	22.2 ± 2.3	19.9 ± 2.9	22.2 ± 2.8	18.6 ± 1.4^**^

Significant difference was found between AGNHP and the corresponding cinnabar dose groups (^*^ *p* < 0.05, ^**^ *p* < 0.01, ^****^ *p* < 0.0001).

Consistent with a previous study, cinnabar with herbal ingredients combination promoted the absorption of mercury and prolonged the elimination process (Lu et al., 2020). A three-year-old boy was diagnosed with mercury intoxication with high blood mercury levels detected in a private laboratory (Valido et al., 2019). The blood levels of mercury above 100 ng/ml were diagnosed with poisoning (Kamensky et al., 2019). After 28 days of exposure to methylmercury, the blood mercury concentration was 76.4 μg/ml, without apparent toxicity observed in rats (Pelletier et al., 2019). Although AGNHP significantly increased blood mercury exposure, it was proved safe by the limited blood mercury exposure at clinical doses. A California woman was diagnosed with mercury poisoning by using a skin-lightening cream on her face twice a day for 7 years, and the contents of mercury were 2,620 and 110 μg/L in her blood and urine samples, respectively (Kuehn 2020). Because mercury absorption through the skin can even lead to poisoning, long-term or excessive usage of cinnabar-containing TCMs should be paid more attention.

The pharmacokinetic profiles of arsenic and mercury in AGNHP were comprehensively elucidated at the first time. The blood exposure of arsenic and mercury were all within the safe range, and no noticeable toxic effects were observed at the experimental dose range of AGNHP. However, long-term or overdose of realgar- and cinnabar-containing TCMs may pose health risks of poisoning due to the slow absorption and elimination of arsenic and mercury.

### Urinary excretion

**FIGURE 3:**
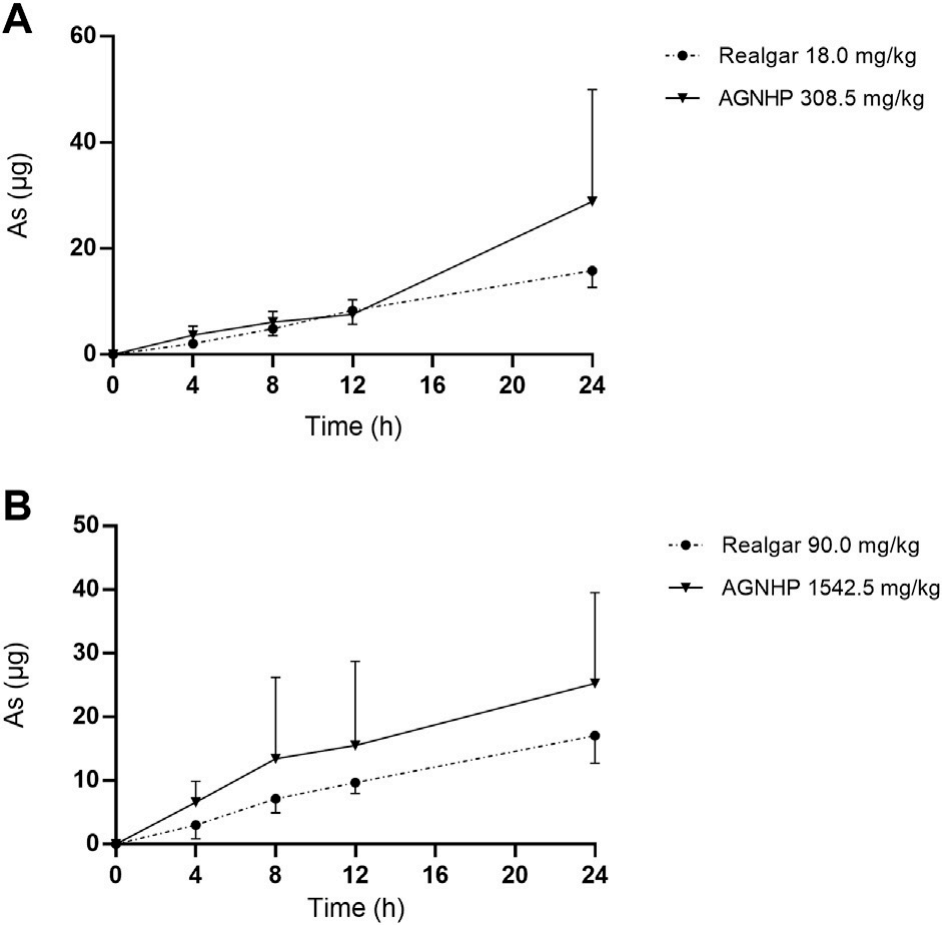
Cumulative excretion of arsenic in rat urine after oral administration of realgar and AGNHP.

**FIGURE 4:**
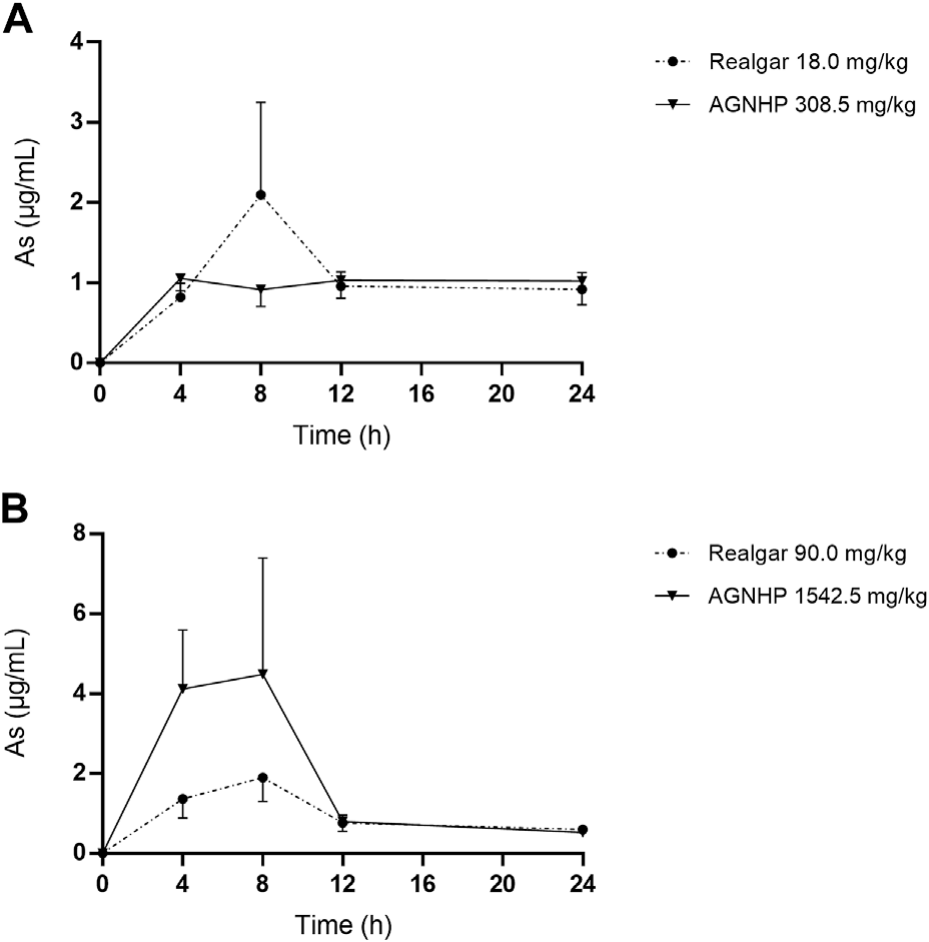
Concentration-time curves of arsenic in rat urine after oral administration of realgar and AGNHP.

The cumulative excretion of arsenic in rat urine after oral administration of realgar and AGNHP is shown in Figure 3. No significant differences were observed in urinary cumulative excretion of arsenic as the increase of dose in realgar and AGNHP groups, respectively. However, the cumulative urinary excretion of arsenic in AGNHP groups was much higher than the corresponding realgar groups. The compatibility of AGNHP promoted urinary arsenic excretion compared with realgar. As shown in Figure 4, the urinary arsenic concentration raised to the maximum at 8 h and then decreased within 24 h. The urinary arsenic concentration of AGNHP did not show noticeable differences between realgar at low doses due to the limited arsenic exposure. However, it was much higher than realgar at high doses. The urinary arsenic was not thoroughly eliminated within 24 h, which was in agreement with our previously reported (Wu et al., 2022). The total urinary cumulative excretion rates of arsenic in realgar and AGNHP low-dose groups were 0.57 and 1.0%, respectively. Meanwhile, those in high-dose groups were 0.12 and 0.18%, respectively. The extremely low urinary excretion rate of arsenic revealed that urine was not the dominant pathway of arsenic.

**FIGURE 5:**
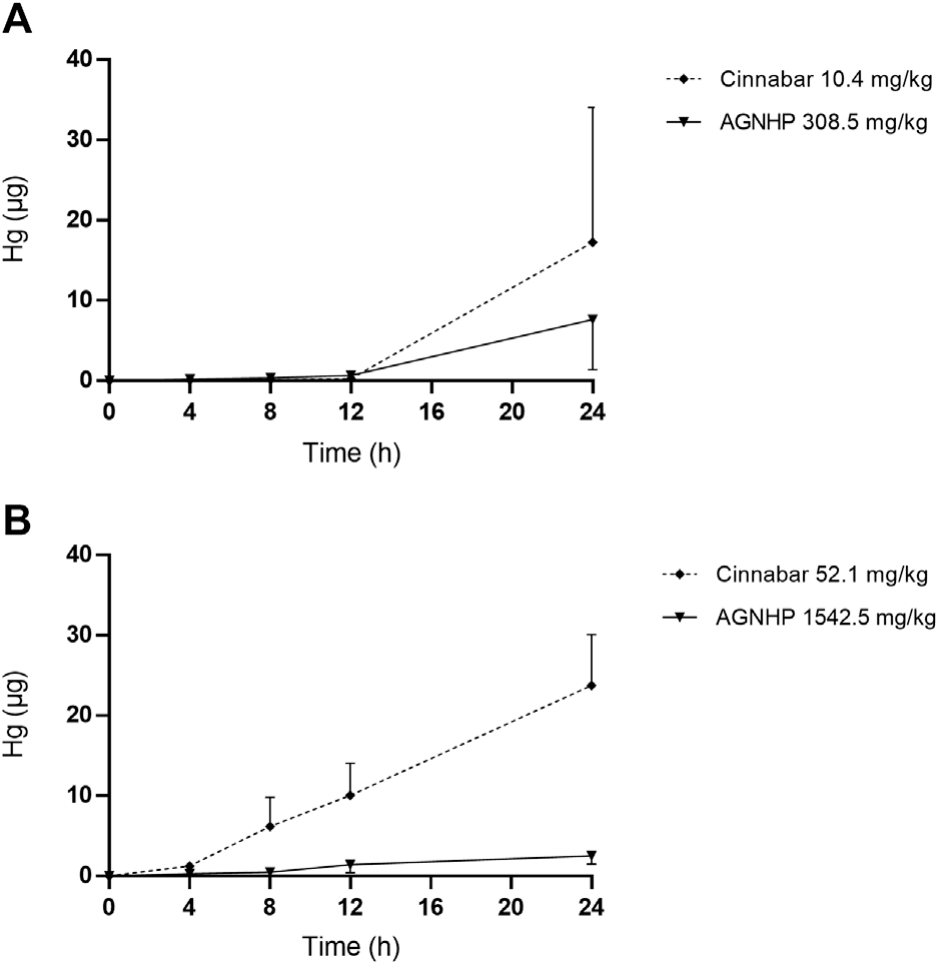
Cumulative excretion of mercury in rat urine after oral administration of cinnabar and AGNHP.

**FIGURE 6:**
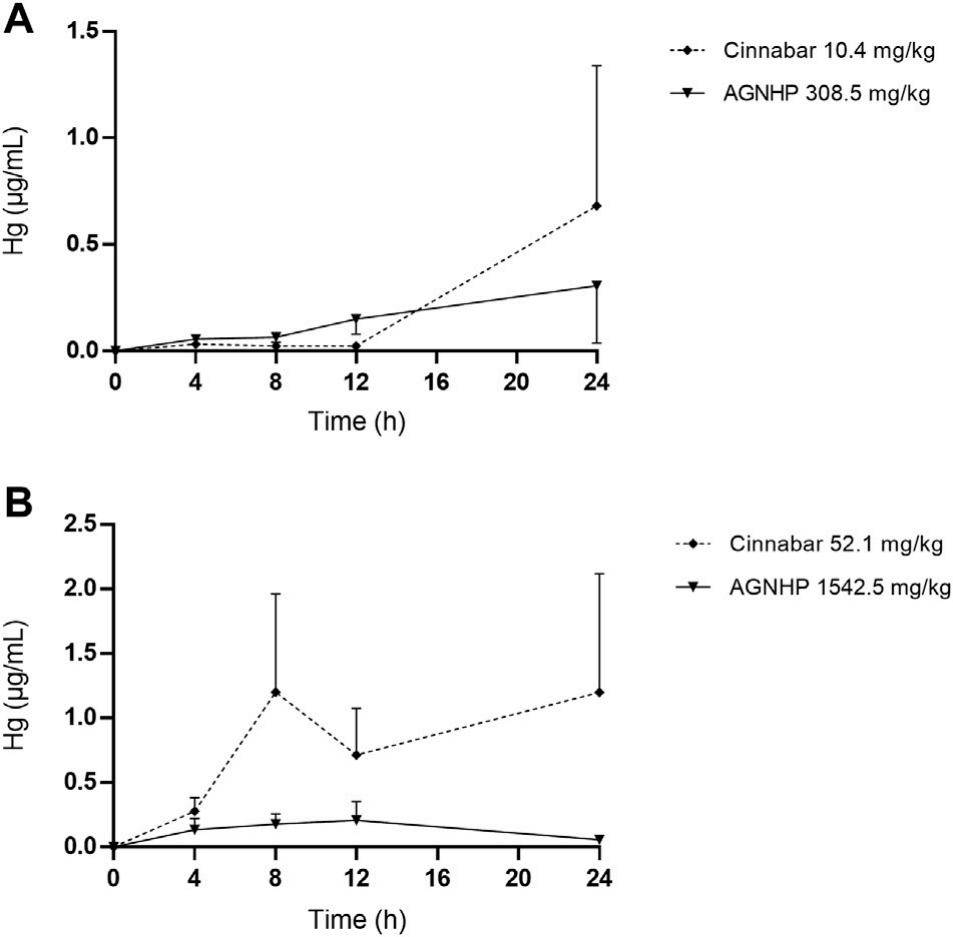
Concentration-time curves of mercury in rat urine after oral administration of cinnabar and AGNHP.

The cumulative excretion of mercury in rat urine after oral administration of cinnabar and AGNHP is displayed in Figure 5. No significant differences were observed in urinary cumulative excretion of mercury as the increase of dose in cinnabar and AGNHP groups, respectively. Interestingly, the cumulative excretion of mercury in rat urine was opposite to that of arsenic after AGNHP compatibility. The cumulative urinary excretion of mercury in AGNHP was significantly reduced compared with the corresponding cinnabar groups. As displayed in Figure 6, the urinary concentration of mercury was much lower than arsenic. The urinary mercury concentration was gradually increased to 24 h after administration at low doses. However, it increased to the maximum at 12 h and then reduced within 24 h after 1542.5 mg/kg AGNHP dosing. Additionally, it was not decreased after treatment with 52.1 mg/kg of cinnabar. The trend of mercury urinary concentration was consistent with its cumulative excretion. Compared with cinnabar, urinary mercury was more difficult to excrete after taking AGNHP. The total urinary cumulative excretion rates of mercury in cinnabar and AGNHP low-dose groups were 0.91 and 0.40%, respectively. And those in high-dose groups were 0.25 and 0.03%, respectively. The results indicated that the elimination of mercury in urine was more difficult than arsenic after AGNHP administration. Urinary mercury was hardly excreted as the dose increases, which may pose a health risk of mercury accumulation by long-term or excessive medication.

The results obtained in urine were in accordance with the findings of blood as we noted above. AGNHP formula compatibility significantly increased the excretion of arsenic in urine and reduced its exposure in blood. Conversely, AGNHP significantly reduced the excretion of mercury in urine and increased its exposure in blood. Urinary excretion was not the dominant excretion pathway of arsenic and mercury, and most of them excreted in feces in the form of As_2_S_2_ and HgS without metabolic transformation (Wu et al., 2022; Zhao et al., 2022). As reported, realgar-containing TCMs significantly inhibited the absorption of arsenic and promoted its excretion, which was responsible for the toxicity-reducing effect (Xu et al., 2022). More than 90.20 and 82.83% of mercury were excreted in rat feces within 24 h after treatment of cinnabar and AGNHP, respectively (Li et al., 2006). Others have also confirmed that cinnabar-containing TCMs significantly reduced the fecal mercury compared with cinnabar (Tian et al., 2015). These findings were highly consistent with our results. AGNHP significantly increased the excretion of arsenic, and conversely reduced the excretion of mercury. Although the fecal excretion profiles of arsenic and mercury have been generally recognized after treatment of realgar- or cinnabar-containing TCMs (Liu et al., 2008; Tinggi et al., 2016; Liu et al., 2018), little is known about the exposure of bioaccessible arsenic and mercury *in vivo*. Consequently, we focused on the bioaccessible arsenic and mercury in rat blood and urine to evaluate the safety of AGNHP in this study.

It has been demonstrated that the normal level of urinary arsenic was less than 0.02 μg/ml (Lech and Trela 2005; Spilchuk and Thompson 2019). Urinary mercury should not exceed 20 μg within 24 h, and more than 150 μg was potentially toxic (Nayfeh et al., 2018). The U.S. Federal Biological Exposure Index of mercury was 50 μg/L in urine (Kamensky et al., 2019). A clinical case reported patients with urinary mercury concentrations of 4,828 μg/L or 458 nmol/L were diagnosed with poisoning (Lu Q. Y. et al., 2017). No related toxicity occurred in 30 days realgar treated rats, and the urinary arsenic concentrations were 10.30 and 3.48 μg/ml at 1–12 and 12–24 h, respectively (Yi et al., 2019; Yi et al., 2020). Rats exposed to methylmercury for 9 weeks with a urinary mercury concentration of 3.20 μg/ml revealed no evidence of overt toxicity (Pingree et al., 2001). The urinary concentrations of arsenic and mercury were in the safe range after oral administration of AGNHP, and there were no significant toxic effects. Urine was not the main excretion route for arsenic and mercury (Wu et al., 2022; Zhao et al., 2022), and the limited excretion of arsenic and mercury could not accurately reflect the toxic exposure *in vivo*. Hence, the diagnosis of poisoning should be combined with the concentrations of arsenic and mercury in blood and urine.

## Conclusion

In the present work, comparative pharmacokinetics and urinary excretion of arsenic and mercury were conducted in rats after oral administration of realgar, cinnabar and AGNHP, respectively. AGNHP significantly reduced the absorption of arsenic in blood and increased the excretion of arsenic in urine. Whereas, the trend of mercury in blood and urine was opposite to that of arsenic after AGNHP administration. AGNHP is safe at clinical doses by the limited arsenic and mercury exposure, but there is still a risk of toxicity by long-term or excessive medication. Therefore, more attentions must be paid to realgar- and cinnabar-containing TCMs due to the slow accumulation and excretion of arsenic and mercury.

## Data Availability

The raw data supporting the conclusion of this article will be made available by the authors, without undue reservation.
